# Advanced GeSn/SiGeSn Group IV Heterostructure Lasers

**DOI:** 10.1002/advs.201700955

**Published:** 2018-03-27

**Authors:** Nils von den Driesch, Daniela Stange, Denis Rainko, Ivan Povstugar, Peter Zaumseil, Giovanni Capellini, Thomas Schröder, Thibaud Denneulin, Zoran Ikonic, Jean‐Michel Hartmann, Hans Sigg, Siegfried Mantl, Detlev Grützmacher, Dan Buca

**Affiliations:** ^1^ Peter Grünberg Institute 9 (PGI‐9) and JARA‐Fundamentals of Future Information Technologies (JARA‐FIT) Forschungszentrum Jülich 52425 Jülich Germany; ^2^ Central Institute for Engineering, Electronics and Analytics Forschungszentrum Jülich 52425 Jülich Germany; ^3^ IHP 15236 Frankfurt (Oder) Germany; ^4^ Department of Sciences Università Roma Tre 00154 Rome Italy; ^5^ CEMES CNRS 31055 Toulouse France; ^6^ Ernst Ruska‐Centre for Microscopy and Spectroscopy with Electrons and Peter Grünberg Institute 5 (PGI‐5) Forschungszentrum Jülich 52425 Jülich Germany; ^7^ Institute of Microwaves and Photonics School of Electronic and Electrical Engineering University of Leeds Leeds LS2 9JT UK; ^8^ CEA LETI MINATEC Campus F‐38054 Grenoble France; ^9^ Université Grenoble Alpes F‐38000 Grenoble France; ^10^ Laboratory for Micro‐ and Nanotechnology (LMN) Paul Scherrer Institute CH ‐5232 Villigen Switzerland

**Keywords:** GeSn, heterostructures, lasers, multi‐quantum wells, SiGeSn

## Abstract

Growth and characterization of advanced group IV semiconductor materials with CMOS‐compatible applications are demonstrated, both in photonics. The investigated GeSn/SiGeSn heterostructures combine direct bandgap GeSn active layers with indirect gap ternary SiGeSn claddings, a design proven its worth already decades ago in the III–V material system. Different types of double heterostructures and multi‐quantum wells (MQWs) are epitaxially grown with varying well thicknesses and barriers. The retaining high material quality of those complex structures is probed by advanced characterization methods, such as atom probe tomography and dark‐field electron holography to extract composition parameters and strain, used further for band structure calculations. Special emphasis is put on the impact of carrier confinement and quantization effects, evaluated by photoluminescence and validated by theoretical calculations. As shown, particularly MQW heterostructures promise the highest potential for efficient next generation complementary metal‐oxide‐semiconductor (CMOS)‐compatible group IV lasers.

The ever‐increasing demand for computational power, e.g., in data centers or consumer electronics, requires new approaches tackling energy efficiency of computer chips. Optical short‐range and on‐chip communication may here offer dramatically reduced power dissipation.^1^ In this respect, Group IV photonics is the key technology,^2^, ^3^ allowing seamless integration of complementary metal‐oxide‐semiconductor (CMOS)‐based electronics with optical components.^4^, ^5^ Since the indirect nature of their bandgap hinders Si and Ge from acting as efficient light emitters, heterogeneous integration^6^ and epitaxy of III–V materials on Si are the solutions presently investigated, yielding optically^7^ and electrically driven^8^, ^9^ lasers. Recently, solutions based on pure group IV alloys got renewed interest as incorporation of group IV element tin (Sn) in Ge and SiGe has led to (i) the formation of truly Si‐compatible, direct gap GeSn^10^ and SiGeSn^11^ systems and, (ii) several demonstrations of GeSn optically pumped lasing.^10^, ^12^, ^13^, ^14^ Moreover, their bandgap in the mid‐infrared range turns them into key materials for biological and chemical sensing applications.^3^ Electrically induced luminescence has been, however, so far demonstrated only for homojunction diodes and/or for indirect gap GeSn structures.^15^, ^16^, ^17^, ^18^

Lessons learned from highly mature III–V laser materials can serve as a model for developing more efficient group IV light emitters, e.g., by introduction of heterostructures or utilizing quantum size effects in the active region.^19^ In this respect, larger‐bandgap SiGeSn ternaries appear to be natural candidates for heterostructures.^20^, ^21^ The use of Sn‐based heterostructures and quantum wells combining binaries and ternaries has been theoretically proposed for energy efficient electronic devices,^22^ photonic applications,^20^, ^21^ or photovoltaics.^23^ Epitaxial growth and characterization were, however, mostly limited to bulk layers^11^, ^24^, ^25^ or single wells.^26^ Recently, its epitaxial integration for GeSn/SiGeSn heterostructure light emitting diodes (LEDs) has been demonstrated,^11^ which significantly improved emission characteristics compared to bulk homojunction LEDs.^27^ However, no combination of SiGeSn with direct bandgap GeSn has been demonstrated yet, which will be the key for any efficient light emitter.

In the present study, we report on growth and characterization of various types of GeSn/SiGeSn heterostructures, combining for the first time relaxed direct bandgap GeSn active layers with SiGeSn barriers. Based on our theoretical models and experimental photoluminescence (PL) and lasing data, the presented work gives a promising perspective for group IV heterostructure lasers, paving the way toward integrated emitters on Si platform.

The heterostructures investigated here make use of buffer technology. A partially relaxed 200 nm thick lower Sn content Ge_0.9_Sn_0.1_ layer is used as buffer. It reduces the lattice mismatch between the Ge virtual substrate (Ge‐VS) underneath and the top Ge_1−_ *_x_*Sn*_x_* active layer, allowing higher Sn incorporation (*x* > 10%) and diminished compressive strain. As those properties increase the energy difference between the indirect L‐ and the direct Γ‐valley, later termed directness, emission properties shall be strongly enhanced in the active region.^28^

The first investigated heterostructure type are double heterostructures (DHSs), where a highly strain relaxed GeSn active layer with high Sn incorporation of about 14 at%, sandwiched between two SiGeSn barrier layers, is targeted. A straightforward way to increase Sn incorporation in GeSn alloys, while maintaining high layer quality, is to decrease growth temperature.^29^ This, on the other hand, limits the amount of incorporable Si in the SiGeSn cladding layers due to the limited disilane precursor cracking.^11^ Large amount of Si incorporation is, however, required for high band offsets to the active region. Moreover, due to the metastable nature of Sn‐based alloys, optimizing the thermal budget during growth is utterly important.^30^, ^31^ Therefore, growth methodology is crucial. Two different approaches were used for the DHS stacks (see **Table** **1**). For the first one, consequently labeled DHS1, the GeSn buffer and bottom SiGeSn cladding were grown in one step at a temperature of 360 °C. Then, the reactor was cooled to 350 °C for growth of the active GeSn region and the top SiGeSn cladding layer. For the second structure, labeled DHS2, the temperature reduction occurred directly after buffer growth, so that the complete SiGeSn/GeSn/SiGeSn DHS stack was grown at 350 °C. Thus, the main difference between the two structures is the stoichiometry of the bottom SiGeSn layers, which was extracted to a Si/Sn incorporation of 5.5/11.5 and 5.5/13 at% for DHS 1 and DHS2, respectively. Details can be found in the Experimental Section and Table 1. For DHS1, this difference leads to better carrier confinement both for L‐ and especially for Γ‐electrons (110 meV for DHS1 vs 47 meV DHS2), which is shown later in Figure 3a.

**Table 1:** Overview on the main structural properties of the grown heterostructures. Except for the bulk GeSn layer, all heterostructure samples feature a 200 nm thick partially relaxed Ge_0.9_Sn_0.1_ buffer

Name	Sn content in active regiona) [nm]	GeSn active regiona) thickness [nm]	Si/Sn content in top cladding [at%]	Si/Sn content in bottom cladding [at%]	Si/Sn content in barrier [at%]	Barrier thickness [nm]
DHS1	14.5	377	4.5/14.0	5.5/11.5	–	–
DHS2	14.0	342	5.0/13.5	5.5/13	–	–
MQW1	13.3	10 × 22	–	–	4.8/13.0	22
MQW2	13.5	10 × 12	–	–	5.2/13.4	16
Bulk GeSn	12.5	414	–	–	–	–

^a)^ Well regions in case of the MQW structures.

X‐ray diffraction reciprocal space maps (XRD‐RSMs) indicate that the Ge_0.9_Sn_0.1_ buffers are partly relaxed with a residual compressive strain of only −0.4% (shown for DHS1 in **Figure** **1**a). Both cladding layers are, if at all, hardly distinguishable in the RSM. The bottom SiGeSn cladding layer's signal largely superimposes the one from the GeSn buffer. The top SiGeSn layer is grown coherently on top of the active GeSn, and can be linked to a signal with the same in‐plane reciprocal lattice constant *Q_x_* (see Figure 1a). The active Ge_0.855_Sn_0.145_ layer in DHS1 and Ge_0.86_Sn_0.14_ layer in DHS2 are partly relaxed with respect to the Ge_0.90_Sn_0.10_ buffers, being under compressive strain of −0.56% and −0.70%, respectively. Transmission electron microscopy (TEM) analysis (Figure 1b) indicates the formation of additional unwanted misfit dislocations at the bottom SiGeSn/active GeSn interface, some of which are marked by white arrows. Apart from that, very homogeneous incorporation of Sn atoms throughout the structure is evidenced by the overlaid secondary ion mass spectrometry (SIMS) depth profile, showing that Si atoms are present only in the cladding regions.

**Figure 1:**
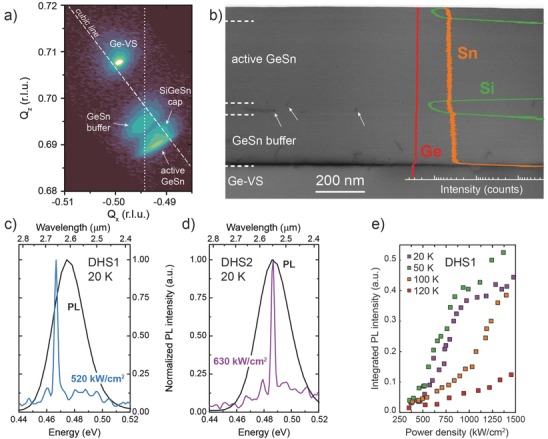
a) XRD‐RSM of DHS (DHS1). b) Cross‐sectional TEM image of a SiGeSn/GeSn/SiGeSn DHS (DHS2), the different layers can be distinguished by the SIMS spectra. Normalized PL and laser emission spectra at 20 K for c) DHS1 and d) DHS2. e) Temperature‐dependent LL curves for DHS1.

Microdisk lasers were fabricated according to the procedure reported in ref. 12 to study the influence of confinement and misfit dislocations on lasing characteristics, such as threshold and maximum operating temperature. Both the heterostructures show lasing under 1550 nm wavelength optical pumping at 20 K, as depicted in Figure 1c,d in comparison to PL. The difference in PL emission wavelength of both the structures can be attributed to small Sn variations, while the shift of lasing emission is a consequence of both band filling (blueshift) and layer relaxation in underetched microdisks (redshift).

This first demonstration of optically pumped lasing in group IV heterostructures discloses two important findings. First and foremost, lasing is achieved with SiGeSn ternaries as barriers to form heterostructures. As shown in Figure 1e, however, lasing ceases at temperatures above 120 K, where the linearity of the light‐in light‐out (LL) curve throughout all pumping levels can be taken as a sign of spontaneous emission dominated luminescence. This is linked to increased losses from nonradiative recombination through defects or Γ–L intervalley scattering, which cannot be overcome by the material gain anymore.

In comparison to laser structures reported earlier in literature,^12^, ^14^ introduction of different SiGeSn claddings for carrier confinement does not result in improved lasing thresholds. Their theoretical advantage seems to be shadowed by a common drawback of both structures: a newly formed misfit dislocation network at the GeSn/SiGeSn interface, making the structures behave similar to bulk GeSn layers. This major drawback can be overcome by using strain‐adjusted heterostructures, such as GeSn/SiGeSn multi‐quantum wells (MQWs), as we show in the following text.

Two such heterostructures, MQW1 and MQW2 (see Table 1), each containing ten periods of GeSn wells and SiGeSn barriers, were grown on top of a partially relaxed Ge_0.9_Sn_0.1_ buffer, similar to the DHS samples. XRD and depth‐calibrated SIMS measurements (not shown here) yield well thicknesses of 22 and 12 nm, respectively. Due to the complexity of the MQW stack, XRD measurements can deliver only average compositional information of the superlattices. Instead, high resolution elemental analysis was performed by means of atom probe tomography (APT). The reconstructed Si atom positions for MQW1 are shown in **Figure** **2**a. Si atoms are strictly confined within the SiGeSn barriers, with only 0.03 at% found in the well region. Elemental profiles (Figure 2b) passing across six central wells, indicate a Sn content of 13.3 at% in the wells, while Si and Sn incorporation of 4.8 and 13.0 at%, respectively, are extracted for the SiGeSn barriers.

**Figure 2:**
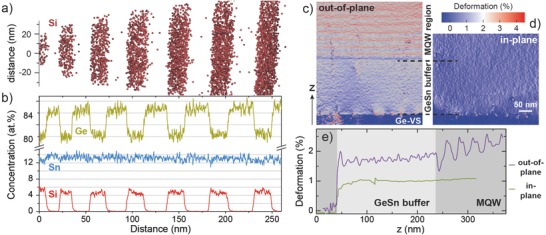
a) Si elemental map and b) concentration profiles across wells and barriers from atom probe tomography analysis of a GeSn/SiGeSn MQW structure. c–e) Out‐of‐plane/in‐plane deformation and both the profiles from TEM holography analysis.

The distinct advantage of the MQW structure is revealed by dark‐field electron holography measurements (MQW2 shown here). Due to its high spatial resolution of about 6 nm, in‐ and out‐of‐plane deformations can be locally resolved. Colored lattice deformation maps reflect lattice strain differences for out‐of‐plane (Figure 2c) and in‐plane (Figure 2d) directions. The constant in‐plane deformation, obvious in the line scans through buffer and MQW in Figure 2e, proves coherent growth of the MQW on top of the Ge_0.9_Sn_0.1_ buffer. This should greatly enhance light emission, as the nonradiative recombination of carriers at the bottom interface with the buffer can be eliminated.^32^

In **Figure** **3**a–c, the calculated conduction band energies in DHS1, DHS2, MQW1, and MQW2 heterostructures are shown. Solid lines represent the lowest bulk conduction band energy, and dotted lines denote the first quantized electron state at Γ. For easier comparison, the displayed conduction band and state energies are measured from the top valence band energy, which in case of the MQW structures is the first quantized heavy hole state. Normalized low temperature PL is juxtaposed in Figure 3d–f to illustrate the effect of quantum confinement in the Ge_0.867_Sn_0.133_ wells. For DHS2, the predicted bandgap of 0.474 eV closely fits PL emission at about 0.485 eV. A large blueshift of PL emission for the MQW heterostructure with 22 nm well thickness can be observed Figure 3e, which is attributed to a slightly smaller Sn incorporation (13.3 at% instead of 14 at% Sn) and to level splitting because of quantization. The additional blueshift of 20 meV for the MQW2 sample with 12 nm thin wells (Figure 3f) is solely a quantum confinement effect, as indicated by band structure calculations. Thus, clear emission from quantized states is observed in the MQW heterostructures.

**Figure 3:**
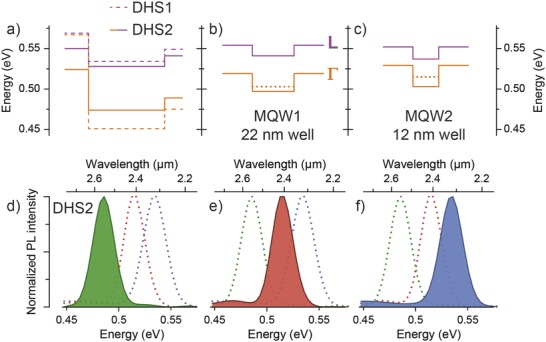
Conduction band alignment (and the lowest quantized states positions) for a) DHS1 and DHS2 and MQW samples with b) 22 nm well thickness (MQW1) and c) 12 nm well thickness (MQW2) at 4 K. d–f) The corresponding PL emission illustrates a peak shift, in line with bandgap changes.

Temperature‐dependent PL emission between 4 K and room temperature offers additional information on carrier confinement. As shown in **Figure** **4**a,b, DHS1 shows only a marginally increased PL signal at cryogenic temperatures compared to a 400 nm bulk GeSn layer with 12.5 at% Sn. A different picture emerges for the 22 nm MQW heterostructure (MQW1) in Figure 4c. Coherent growth leads to a strong increase of PL at low temperatures (an order of magnitude at 4 K). This distinct advantage is expected to result from both the confinement of carriers in the GeSn wells and their spatial separation from the GeSn/Ge–VS defective interface. Such a heterostructure is expected to improve lasing performance, such as the lasing threshold, which will be discussed in more details elsewhere. However, too strong quantization, as present in MQW2, diminishes the energy difference between Γ‐ and L‐valleys due to the smaller effective mass of Γ‐electrons in comparison to L.^16^ In this case, the decreased directness (and even transition to indirect semiconductor for thinnest wells) leads to poor light emission (98% less PL emission at 4 K compared to MQW1).

**Figure 4:**
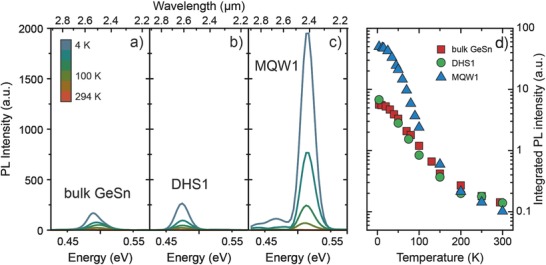
Temperature‐dependent PL emission for a) bulk GeSn, b) a double heterostructure, and c) a multi‐quantum well with 22 nm well thickness. d) Integrated PL intensity shows the strongest emission from MQW1 at cryogenic temperatures.

The above described PL effects are quantified in a logarithmic plot of the integrated PL intensity in Figure 4d. The first observation is that DHS1 yields no light emission improvement over a bulk GeSn sample (see Table 1), as both are limited by the above mentioned misfit dislocation network at the GeSn/SiGeSn interface. In contrast, MQW1 shows the strongest PL emission of the investigated samples, at least at cryogenic temperatures, hinting to a considerably increased nonradiative lifetime. The particular advantage of MQW1 in terms of light emission, however, disappears for temperatures above ≈150 K. This can be related to the relatively low conduction band offset between wells and barriers (13 meV determined by band structure calculations). Above this temperature, carriers are able to overcome the energetic barriers and nonradiative recombination at the GeSn/Ge interface sets in. In fact, at room temperature, the integrated MQW1 PL emission intensity is below the one of DHS1 and the bulk GeSn sample, which we attribute to the thinner optically active layers. Therefore, we can deduce that coherent growth, i.e., epitaxy schemes avoiding the formation of additional misfit dislocations, is the most vital pathway toward further improvement of the optical properties. For example, growth of thinner active regions and/or optimized GeSn buffers will be developed. Moreover, to effectively screen carriers from nonradiative recombination sinks at the bottom GeSn/Ge interface, we consider growing SiGeSn barriers with higher Si content. In that case, we expect a distinct advantage of MQW heterostructures, compared to double heterostructures and even more bulk GeSn layers. These features may allow room temperature, low threshold lasers made entirely from group IV materials.

In summary, we demonstrated the potential of group IV epitaxy for the fabrication of direct bandgap heterostructures. Different designs, from bulk to double heterostructures and multi‐quantum wells, were epitaxially grown and characterized. A high sample quality is proven by the first demonstration of optically pumped lasing from group IV GeSn/SiGeSn heterostructures. For the MQW heterostructures, a shift in light emission is achieved by controlling the quantum confinement of carriers through varying well thicknesses. But most importantly, this type of structure outperforms double heterostructures, because of the spatial separation of misfit dislocations from the active region. We indicated that by increasing the heterostructure barriers, the favorable MQW emission properties can be extended to higher temperatures, possibly up to room temperature. In conclusion, we believe that, as demonstrated decades ago already for the III–V material system, group IV heterostructures and multi‐quantum wells are potential enablers for high efficient integrated light emitters.

## Experimental Section

*Epitaxy*: All investigated layers were produced by means of chemical vapor deposition (CVD) in an industry‐compatible reactor design on 200 mm wafers. The employed precursors disilane (Si_2_H_6_), digermane (Ge_2_H_6_), and tin tetrachloride (SnCl_4_) ensure high growth rates (typically 15–20 nm min^−1^) at low growth temperatures (between 350 and 360 °C), helpful for suppression of quality‐degrading Sn precipitates and surface segregation. To reduce the large lattice mismatch between the Si (001) and the (Si)GeSn alloys, growth was performed on top of around 2.5 µm thick Ge–VS.^33^ Prior to the growth, the native oxide was removed in an automated single‐wafer cleaning tool using hydrofluoric acid vapor chemistry, followed by an in situ hydrogen bake.

*Material Characterization*: The local material parameters inside the complex heterostructures were determined by a large number of techniques. For the MQWs, exact compositions were obtained using APT, while energy‐dispersive X‐ray spectroscopy (EDX) mapping ensured precise values for the DHS. Layer thicknesses were determined likewise from EDX and validated from XRD (MQW) and SIMS (DHS). Strain values were obtained from XRD reciprocal space maps and validated by dark‐field electron holography maps.

Band structure values at critical energies were obtained at 0 K by the empirical pseudopotential method and appropriate strain correction within an 8‐band k∙p framework. The effect of temperature was included via Varshni's empirical relations. All theoretical parameters, required for modeling the band structure, can be found in ref. 27 and the Supporting Information of ref. 10.

## Conflict of Interest

The authors declare no conflict of interest.
